# DeepDRG: Performance of Artificial Intelligence Model for Real-Time Prediction of Diagnosis-Related Groups

**DOI:** 10.3390/healthcare9121632

**Published:** 2021-11-25

**Authors:** Md. Mohaimenul Islam, Guo-Hung Li, Tahmina Nasrin Poly, Yu-Chuan (Jack) Li

**Affiliations:** 1 Graduate Institute of Biomedical Informatics, College of Medical Science and Technology, Taipei Medical University, Taipei 110, Taiwan; d610106004@tmu.edu.tw (M.M.I.); d610108004@tmu.edu.tw (T.N.P.); 2 AESOP Technology, Songshan District, Taipei 105, Taiwan; jacky@aesoptek.com; 3 International Center for Health Information Technology (ICHIT), Taipei Medical University, Taipei 110, Taiwan; 4 Research Center of Big Data and Meta-Analysis, Wan Fang Hospital, Taipei Medical University, Taipei 116, Taiwan; 5 Department of Dermatology, Wan Fang Hospital, Taipei 116, Taiwan; 6 TMU Research Center of Cancer Translational Medicine, Taipei Medical University, Taipei 110, Taiwan

**Keywords:** deep learning, artificial intelligence, diagnosis-related groups, hospital expenditure

## Abstract

Nowadays, the use of diagnosis-related groups (DRGs) has been increased to claim reimbursement for inpatient care. The overall benefits of using DRGs depend upon the accuracy of clinical coding to obtain reasonable reimbursement. However, the selection of appropriate codes is always challenging and requires professional expertise. The rate of incorrect DRGs is always high due to the heavy workload, poor quality of documentation, and lack of computer assistance. We therefore developed deep learning (DL) models to predict the primary diagnosis for appropriate reimbursement and improving hospital performance. A dataset consisting of 81,486 patients with 128,105 episodes was used for model training and testing. Patients’ age, sex, drugs, diseases, laboratory tests, procedures, and operation history were used as inputs to our multiclass prediction model. Gated recurrent unit (GRU) and artificial neural network (ANN) models were developed to predict 200 primary diagnoses. The performance of the DL models was measured by the area under the receiver operating curve, precision, recall, and F1 score. Of the two DL models, the GRU method, had the best performance in predicting the primary diagnosis (AUC: 0.99, precision: 83.2%, and recall: 66.0%). However, the performance of ANN model for DRGs prediction achieved AUC of 0.99 with a precision of 0.82 and recall of 0.57. The findings of our study show that DL algorithms, especially GRU, can be used to develop DRGs prediction models for identifying primary diagnosis accurately. DeepDRGs would help to claim appropriate financial incentives, enable proper utilization of medical resources, and improve hospital performance.

## 1. Introduction

Healthcare spending has consistently been increasing globally. Inpatient care is one of the most expensive hospital services, accounting for approximately 31% of the total expenditure [1]. With the limited resources and increased complexity, policymakers are facing immense challenges of reducing health care costs while improving financial protections, high-quality care, and lowering out-of-pocket (OOP) costs for people [2]. The Fee-for-service (FFS) is a basic payment system for both national and private hospitals in which all care providers are reimbursed for each service provided [3,4]. To rein in excessive healthcare costs and maintain sustainable procedures for inpatients, prospective payment policies are often implemented to foster risk-sharing between insurers and providers [5,6]. Nowadays, the governments of several countries have already reformed their hospital payment policy by shifting from FFS to diagnosis-related groups (DRGs).

The concept of DRGs has now been widely adopted and become the principal means of reimbursement for inpatient services globally [7]. Previous studies have shown that the implementation of DRGs helped to shorten the length of hospital stays and lower OOP by efficiently allocating hospital resources [8,9]. DRGs, a prospective payment system, are used to effectively classify and combine diseases with similar characteristics into different diagnostics and treatment groups. Indeed, DRGs are set to achieve greater equality of financing based on the homogeneity of the clinical process and the similarity of resource consumption [10]. The diagnostics codes should be accurately matched with DRGs codes to claim actual reimbursement, but this is a time-consuming process and requires expert knowledge to manually retrieve information from patients’ clinical records. Selection of appropriate DRGs codes depends on several factors, such as patients’ comorbidities, complications, treatments, age, discharge status, and the principal diagnoses provided by physicians. Therefore, the quality of DRGs coding is a key factor that influences a hospital’s ability to receive reasonable reimbursement and its overall profits.

DRGs coding errors can influence hospitals’ income, hamper proper planning, and often lead to unfair distributions of resources. Ayub et al. [11] evaluated coding accuracy and its impact on hospital costs, reporting 9.6% miscoding with a total lost billing opportunity of $587,799. Cheng et al. [12] reviewed the causes and consequences of miscoding in a Melbourne tertiary hospital, finding that 16% of the 752 cases audited reflected a DRG change and caused a loss of hospital revenue of nearly AUD 575,300. Furthermore, the incorrect selection of the principal diagnosis accounted for an additional 13% of the DRG changes, which is due to the poor quality of documentation [12]. A previous study demonstrated that the overall rate of incorrect DRGs coding was up to 52%, which may be due to a lack of professional experience, work load, and lack of automation [13].

The widespread application of electronic health records (EHRs) has generated large amounts of patient data and created immense opportunities to predict the primary diagnosis using deep learning (DL). In this study, we developed and validated DL models to predict the primary diagnosis for appropriate reimbursement and improve the quality of care.

## 2. Literature Review

Taiwanese residents have been benefited from the nationwide health-care coverage through the compulsory National Health Insurance (NHI) scheme since 1995 [14]. The introduction of this system has ensured high-quality care, and NHI provides reimbursement for nearly all medical fees [15]. However, given the limited resources, global health care systems are facing immense challenges in responding to burgeoning healthcare expenditures [16,17]. Therefore, several strategies have been implemented to reduce unnecessary costs and minimize financial risks from insurers to providers [5,18]. In 2010, Taiwan introduced the diagnosis-related group (DRG) payment system, aiming to improve efficiency and minimize costs. This DRG payment system has an evident impact on current health care services, including the length of hospital stay and the intensity of inpatients care [19].

Artificial intelligence (AI) has shown great promise in improving patient care and making fruitful clinical decisions [20,21]. The availability of EHRs data has created an opportunity to calculate DRGs and associated costs at the time of admission using AI algorithms. The Taiwan National Health Insurance (NHI) Research Database is one of the largest nationwide population databases in the world, which has been used to produce high-quality research [22,23,24,25,26]. As a result, the NHI database can be used to predict DRGs using AI algorithms to accurate reflecting the costs incurred by hospital treatments and stays. Using two cohorts, a prior study applied a deep learning model to automatically predict DRGs and the corresponding costs [27]. Moreover, another study from Germany examined the effectiveness of statistical machine learning in the early prediction of DRG and resource allocation at a 350-bed hospital [28].

### 2.1. Artificial Neural Networks

**Figure 1:**
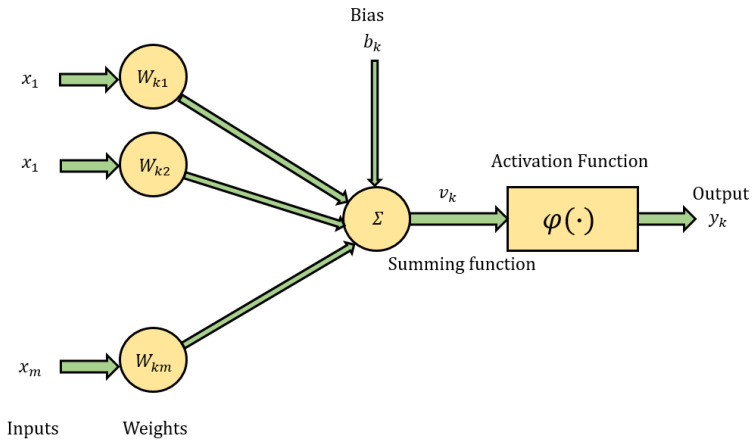
Simple structure of ANN.

Artificial neural networks (ANNs) are also simply called neural networks. ANNs are a subset of machine learning and are at the heart of DL algorithms that are inspired by the biological neural network. The main concept of an ANN was described by McCulloch and Pitts [29] and was finally developed in 1958 [30]. An ANN consists of three layers: the input layer, which receives data, the output layer, which generates valuable information from provided data, and one or multiple hidden layers that are connected to the input and output layer (Figure 1).

For example, each input $x_{i}$ in the input layer is multiplied by a connection weight $w_{ij}$ between the neuron i in the input layer to the neuron j in the hidden layer. However, bias $b_{j}$ is formerly summarized as net input $S_{J}$ and passes it to the hidden layer by a nonlinear activation function, sigma to generate an output, $\;y_{j}$.

$$S_{j}=\sum x_{i}w_{ij}+b_{j}\;\;f\left(S\right)=\frac{\left(1-e^{-2S}\right)}{\left(1+e^{-2S}\right)}\;\;y_{j}=f\left(S_{j}\right)\;$$

However, $y_{i}$ signal from the hidden layer to all k neuron in the final output layer $F_{k}$. and calculate the input to the k neuron of the output layer is $F_{k}^{\prime }$.

$$F_{k}^{\prime }=\overset{h}{\underset{j=1}{\sum }}y_{j}w_{jk}^{\prime }+b_{k}^{\prime }\;$$

where $w_{jk}^{\prime }$ is the weight of the connected between the j neuron in the hidden layer to the k neuron of the output layer; $b_{k}^{\prime }$ is the bias.

Finally, it calculates the output layer signals by using an activation function, sigmoid.

$$F_{k}=f\left(F_{k}^{\prime }\right)\;$$

The error between the target outcome and the observed data is measured as follows:

$$Error_{k}=\frac{1}{2}\overset{n}{\underset{k=1}{\sum }}(target_{k}-observed_{k})\;$$

This process, utilized in all pairs in the training dataset and the training cycles, is known as epoch. The number of epochs is selected by users and repeated to reach minimum error.

The error gradient for output layer is calculated as follows:

$$Gradient\;error_{k}=\left(target_{k}-observed_{k}\right)f^{\prime }\left(F_{k}^{\prime }\right)\;$$

Rosenblatt [30] first proposed the idea of perceptron, which was used in a gradient descent-based learning algorithm. The perceptron is centered on single neuron and can be considered the primary basis of feed-forward ANNs. Perceptron is more generally a computational model, and it takes an input, aggregates it and returns 1 only if the weighted sum is more than some thresholds, otherwise it gives 0. The equation is given below:

$$f\left(net\right)=\{\begin{array}{c} 1\;if\overset{n}{\underset{i=0}{\sum }}w_{i}x_{i}>0\\ 0\;otherwise\;\;\; \end{array}\;$$

However, there were several limitations of using perceptron, which were raised by Minsky and Papert [31], who mentioned that it cannot be used to implement the sample data which are not linearly separable. In the modern era, using backpropagation has immense advantages over traditional gradient descent methods. It provides a way to train networks with any number of hidden units arranged in any number of layers. In fact, the networks do not need to be organized in layers. It provides multi-layer feed-forward ANNs with a highly competitive supervised algorithm. The backpropagation helps to reduce the predefined loss function through updating the weight and bias values [32].

### 2.2. Gated Recurrent Unit

**Figure 2:**
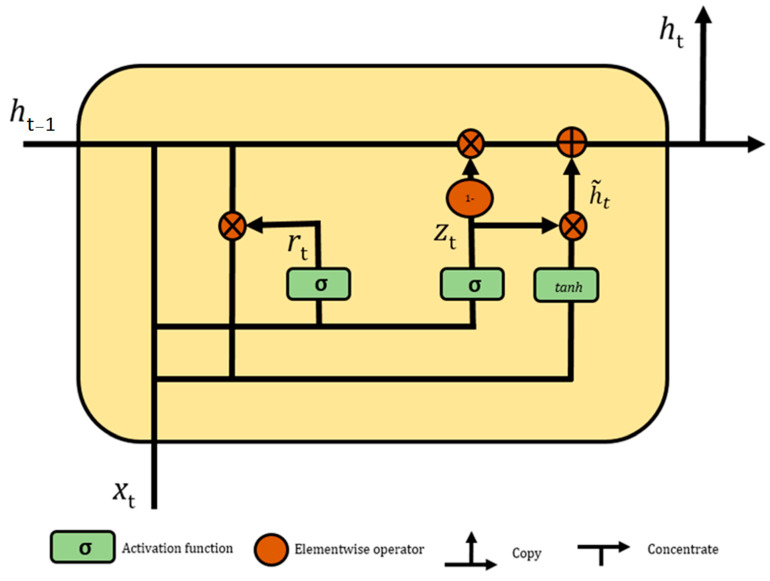
Architecture of gated recurrent unit.

A gated recurrent unit (GRU) uses a gating mechanism in the recurrent neural networks and was first introduced in 2014 by Cho [33]. The GRU is quite similar to long short-term memory (LSTM) with a forget gate, but GRU has only two gates (reset and update,) and LSTM has three gates (input, output, and forget). GRU is less complex, faster, requires less memory and less time to train compared to LSTM because it has less parameters than LSTM. The main advantage of using GRU is that it solves the vanishing gradient problem, which comes with a standard recurrent neural network (RNN). Figure 2 presents the inputs for both the reset and update gates in a GRU where the input of the current time step is $x_{t}$, the hidden state of the previous time step is $h_{t-1}$, and output is calculated by activation function.

Assume that, in a given time step t, the number of provided inputs of a small batch $x_{t}\in {\mathbb{R}}^{n\times h}$ (h is the number of hidden units). Then, the reset gate $r_{t}\in \;{\mathbb{R}}^{n\times h}$ and update gate $z_{t}\in \;{\mathbb{R}}^{n\times h}$ are calculated as follows:

$$r_{t}={\;\sigma }_{GRU}\left(x_{t}w_{xr}+h_{t-1}w_{hr}+b_{r}\right)\;\;z_{t}=\sigma_{GRU}\left(x_{t}w_{xz}+h_{t-1}w_{hz}+b_{z}\right)\;$$

Here, $w_{xr},\;w_{xz}\in \;{\mathbb{R}}^{v\times h}\;\mathrm{and}\;w_{hr},\;w_{hz}\in \;{\mathbb{R}}^{h\times h}$ are weight parameters, $b_{r},\;b_{z}\in \;{\mathbb{R}}^{1\times h}$ are biases, and $\sigma_{GRU\;}$ is an activation function, which converts values in both gates into 0–1.

Later, the candidate hidden state ${\tilde{h}}_{t}\in \;{\mathbb{R}}^{n\times h}$ is generated in a series of operations between the output of the reset gate and the hidden state $h_{t-1}$ of the previous time step. It is calculated as follows:

$${\tilde{h}}_{t}=tanh_{GRU}\left(x_{t}w_{xh}+\left(r_{t}⊗h_{t-1}\right)w_{hh}+b_{h}\right)\;$$

where $w_{xh}\in \;{\mathbb{R}}^{v\times h}\;\mathrm{and}\;w_{hh}\in \;{\mathbb{R}}^{h\times h}$ are weight parameters, $b_{h}\in {\mathbb{R}}^{1\times h}$ is the bias, ⊗ is the elementwise product operator, and $tanh_{GRU}$ is a nonlinear activation function, which converts value between −1 and 1. In this stage, the influence of previous states can be minimized with the elementwise multiplication of $r_{t}\;\mathrm{and}\;h_{t-1}$. The unique part of this equation is how the element value of the reset gate controls how much influence of previous hidden state $h_{t-1}$ can have on the candidate state. If the value of reset gate $r_{t}$ is equal to 1, then all the information from the previous hidden state $h_{t-1}$ is considered. Likewise, if the value of reset gate $r_{t}$ is 0, then the information from the previous hidden state is completely ignored.

After obtaining the result of the candidate state, it is used to generate the current hidden state. This is where the update gate comes on board. However, the equation here is slightly different from LSTM (input and forget gate are complementary and have certain redundancy). GRU directly uses a single update gate to control both historical information, which is the hidden state $h_{t-1}$ of the previous time moment, and the candidate hidden state $\tilde{h}$ of the current time. The final updated equation for the GRU is as follows:

$$h_{t}=z_{t}⊗h_{t-1}+\left(1-z_{t}\right)⊗{\tilde{h}}_{t}\;$$

When the update gate $z_{t}$ is close to 1, it will retain the old state. In this case, the information from $x_{t}$ is completely ignored. When $z_{t}$ is close to 0, then the new latent state $h_{t}$ approaches the candidate latent state ${\tilde{h}}_{t}$.

## 3. Materials and Methods

### 3.1. Study Approval and Propose Methodology

**Figure 3:**
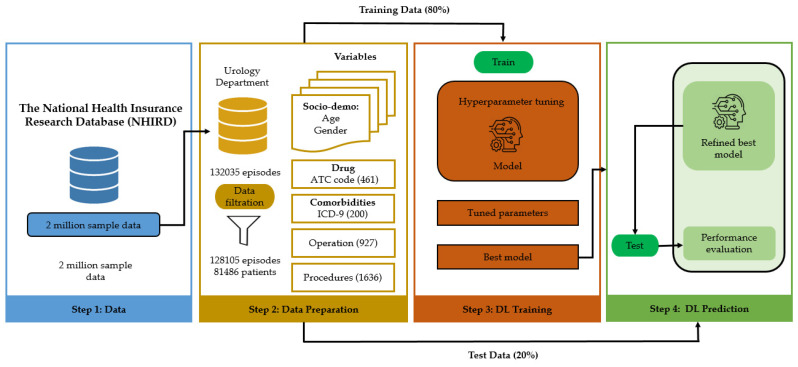
The methodological framework used in this study.

This study was conducted in the multiple center according to the tenets of the Declaration of Helsinki. This study was approved by the Taipei Medical University Institutional Review Board, which waived informed patient consent because all patient records and information were anonymized and deidentified before the analysis. Figure 3 shows the data analysis framework for DRGs prediction in this study.

### 3.2. Data Source

This current study retrieved data from the Taiwan National Health Insurance Research Database (NHIRD) between 1999 and 2013. NHIRD offers the most comprehensive clinical information, such as demographics, diagnosis records, medication records, surgical records, and laboratory information from 23 million people in the Taiwanese population [34,35]. International guidelines were followed to record and collect data, e.g., diseases and medication prescriptions were coded and retrieved by using the International Classification of Diseases, Ninth Revision (ICD-9), and Anatomical Therapeutic Chemical (ATC). The quality and completeness of the NHIRD database is excellent, and it is used to conduct high-quality clinical research. In our study, we collected 2 million random samples from NHIRD. Afterwards, we carried out a retrospective cohort study of individuals who visited the urinary department’s inpatient care between 1999 and 2013. Patients were eligible for inclusion in this study if they were labelled as discharged and had a primary diagnosis for admission, resulting in 132,035 episodes. After filtration (e.g., missing data and infrequent comorbidities), the final study sample included 128,105 episodes from 81,486 patients.

### 3.3. Data Descriptions

In the era of big data, AI models have potential to make advanced clinical decision support and assist clinicians to deliver optimal care. Clinical decision support systems (CDSS) have the ability to analyze large volumes of data and recommend appropriate primary diagnosis for improving efficiency and sustainable care. However, to make a feasible implementation of a DL algorithm to predict DRGs, we included patient and clinical factors readily available in electronic health records (EHRs). More specifically, we collected patients’ sociodemographic, admission status, admission history, admission diagnosis, discharge diagnosis, medications, comorbidities, operations, and procedures history to support real-time, proactive decision making related to selecting appropriate primary diagnosis for effective cost management.

Potential predictor selection was guided by previously published works and data availability. Predictor variables include the following: (i) patient demographics (age, gender), (ii) procedures (1636 types), (iii) drugs (461 types), (iv) operation (927 codes), and (v) the 200 most common comorbidities. Admission and discharge diagnoses were determined using the primary International Classification of Diseases, 9th edition (ICD-9). Moreover, drugs were determined using Anatomical Therapeutic Chemical (ATC), and operation and procedure codes were determined using standard protocol.

### 3.4. Data Preprocessing

Data preprocessing comprised the following steps: (1) data cleaning, (2) variable selection, and (3) one hot coding/embedding formation. NHIRD collects a vast number of variables; however, not all the variables were important to this study. In this process, we deleted irrelevant variables and kept only demographics, admission data, visit date, date of birth, department identification, medications, diseases, operations, laboratory, and procedures information. Afterwards, patients’ age was calculated using their birth date and admission date information. There was no age limit included in our work. For medication information, we used the five-digit Anatomical Therapeutic Chemical Classification System., e.g., five-digit ATC code: B01AC, platelet aggregation inhibitors excluding heparin, including drugs such as B01AC01 (Ditazole), B01AC02 (Cloricromen), and B01AC04 (Clopidogrel). Additionally, seven characters (e.g., A10BA02) were considered for the chemical substances, even though five characters were used to describe the chemical substances, and all the drugs included in this group were prescribed for almost the same purpose. There were 1317 types of primary diagnosis during the study period, but all were infrequent. We therefore calculated the frequency and percentage of primary diagnoses and considered 200 primary diagnoses as our primary outcome. Those 200 diagnoses covered approximately 97% of total diagnoses. The reason for considering the top 200 primary diagnoses as targeted outcomes was because the DL algorithm needs sufficient data to train the model; otherwise, it may perform poorly.

### 3.5. Model Development

**Figure 4:**
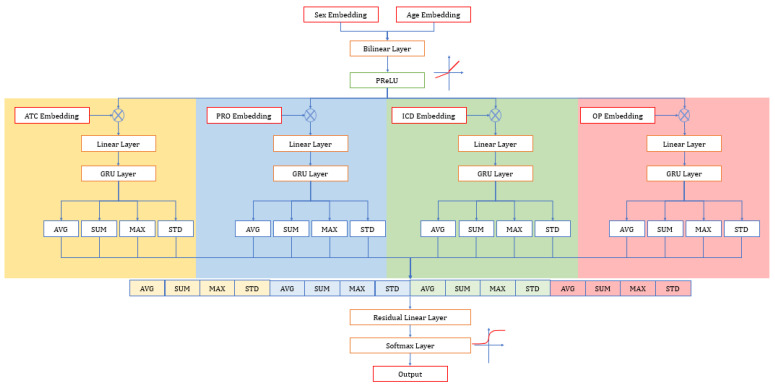
Architecture of GRU model.

We split the data set into a training set (80%) and a testing set (20%). The GRU model was developed to train all the variables, and the model was assessed using the validation set to predict the primary diagnosis. GRU is a high-performing recurrent neural network. It is similar to the LSTM and RNN algorithms, but GRU consists of only two gates—a reset and an update gate [36,37]. The input of GRU moves through the layers, calculating the probability of each output. For the activation function, sigmoid was used in the hidden layers, and Softmax was used in the output layer. The activation function is an integral part of a neural network that is often known for non-linearity, i.e., describing the input and output relations in a non-linear way. The architecture of the current model is shown in Figure 4. Six types of inputs (sex, age, drug, second diagnosis, procedure, and surgery) enter the embedding layer first. Afterwards, the embedding of sex and age enter the bilinear layer and PReLU. The output of the last step is multiplied with other embeddings of inputs. However, the other four are multiplied with embeddings, then enter the linear layers and GRU layers and calculate four statistic values for each output from step 4 and concatenate them. Finally, GRU gives the output as the probability using the activation function and residual layer.

**Figure 5:**
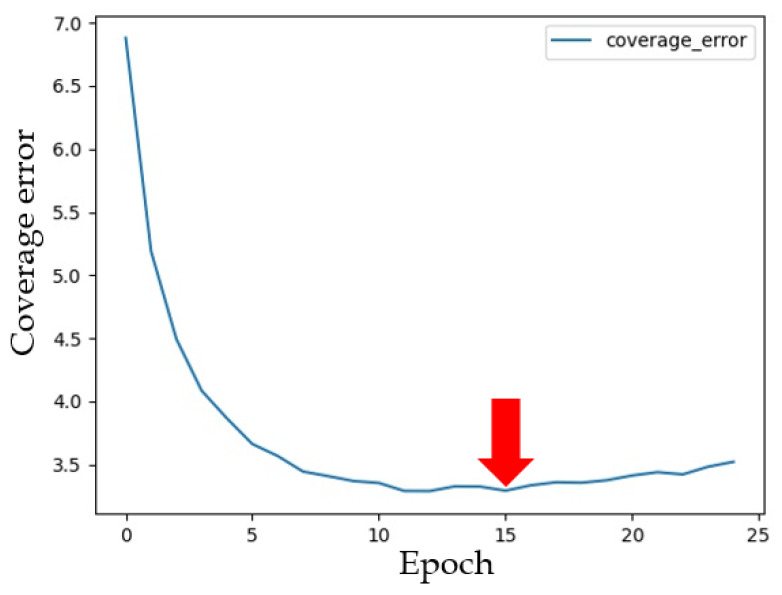
Testing loss of the GRU model.

In our study, we used 25 epochs; however, the model performed well while considering only 15 epochs (Figure 5).

### 3.6. Evaluation Matrices

We evaluated the performance of the DL models on the internal validation set for primary diagnosis recommendations using the following metrics.

Accuracy: It averages the entire set of data as an aggregate result, and calculates 1 metric rather than k metrics.

$$\mathrm{Accuracy}=\frac{\mathrm{TP}+\mathrm{TN}}{\mathrm{TP}+\mathrm{TN}+\mathrm{FP}+\mathrm{FN}}\;$$

where TP = True Positive; TN = True Negative; FP = False Positive, and FN = False Negative.

Micro-F1: It measures the F1-score of the aggregated contributions of all classes. The equation is given below:

$$\mathrm{micro}-F1=2\frac{\mathrm{Micro}-\mathrm{Precision}\;\times \;\mathrm{Micro}-\mathrm{Recall}}{\mathrm{Micro}-\mathrm{Precision}+\mathrm{Micro}-\mathrm{Recall}}\;$$

Micro Precision and Recall: Average precision summarize the fraction of relevant labels ranked higher than the other relevant label. The equation is presented below:

$$\mathrm{Precision}=\frac{\sum_{c}{\mathrm{TP}}_{c}}{\sum_{c}{\mathrm{TP}}_{c}+\sum_{c}{\mathrm{FP}}_{c}}\;\;\mathrm{Recall}=\frac{\sum_{c}{\mathrm{TP}}_{c}}{\sum_{c}{\mathrm{TP}}_{c}+\sum_{c}{\mathrm{FN}}_{c}}\;$$

where c is the class label.

## 4. Results

### 4.1. Patient Characteristics

A total of 128,105 patients admitted to the urinary department were included in this study. There were more male patients than female patients (74.65% vs. 25.35). The number of input and output was 3224 and 200, respectively. Table 1 shows the basic characteristics of patients.

### 4.2. Performance of Deep Learning Model

Table 2 shows the measurement of diagnostic performance (precision, recall, F1-score, accuracy, and AUROC) used to predict the primary diagnosis. The GRU model predicted the primary diagnosis with 83% precision, 66% recall, and 73% F1-score. The ANN model predicted the primary diagnosis with 82% precision, 57% recall, and 67% F1-score. However, the GRU performed better than ANN in predicting the primary diagnosis.

### 4.3. Sensitivity Analysis

In our study, we also tested our model using different numbers of variables and achieved high performance when using all the variables (basic information, drugs, procedures, operations, and additional disease information). However, the model performed worse when only basic information was used. Table 3 shows the performance of the GRU model using different numbers of variables.

### 4.4. Evaluation

After developing and internally validating our model, we evaluated its effectiveness using several unknown cases. Table 4 shows five examples of how our model gave suggestions based on the patients’ inputs. Our model gave the top five recommendations for primary diagnosis, from which the doctor could choose. If the doctor had wanted to see more recommendations, our system would have been able to provide more based on user inputs. However, the top diagnosis was supported by strong evidence. For example, the original primary diagnosis for patient #1 was calculus of ureter, and our model predicted the same primary diagnosis. However, our model will provide additional suggestions for selecting primary diagnosis, and doctor can choose another as either a primary or secondary diagnosis. For patient#4, the original diagnosis was “Malignant bladder neoplasm, other specified sites”, and our model suggested “Malignant bladder neoplasm, part unspecified”. However, the original diagnosis was in our suggested lists, and the physician could have chosen any one of them. The main advantage of using a DL-based system is the availability of a list of appropriate suggestions without manual examination of patients’ documentations.

## 5. Discussion

To the best of our knowledge, this is the first study to examine the performance of a DL model used for the prediction of accurate primary diagnosis. This study shows the rigorous training and testing of a novel deep learning model that has been shown to achieve a high accuracy for the prediction of DRGs. The rapid rise of AI in healthcare offers great opportunities to overcome DRGs’ coding limitations and to claim appropriate reimbursement for inpatient care. The satisfactory performance of DL models using a huge amount of EHRs data can now be provided as an alternative option to traditional manual DRGs coding.

The effectiveness of using DRGs in inpatient care is widely assessed because it is considered as the standard payment management system [38]. The main purpose of using DRGs in the inpatient care setting are: (a) to improve the transparency of the service provided by hospitals, (b) to utilize hospital resources efficiently, and (c) to obtain appropriate reimbursement. Hospital expenditure has increased, and the quality of care has diminished due to improper use of the current DRGs. A study from Saudi Arabia reported a coding error rate of approximately 30% for DRGs [39], while a 51% overall coding error rate was reported in a study from the UK [40]. However, incorrect selection of the primary diagnosis accounted for more than 13% of the DRG changes, and missing additional diagnosis codes accounted for 29% [12]. Moreover, Zafirah et al. reported [41] that the error rate of primary diagnoses was approximately 50%. In addition, the coding error rate of secondary diagnoses was also higher in Malaysia (81.3%), Saudi Arabia (35.6%), and Thailand (28.0%) [39,42,43].

If the DRGs’ reimbursement is not correctly defined, then hospitals will lose income. A previous study reported that a potential loss of hospital income due to coding errors was equivalent to 39.1% of the total hospital income [42]. Maryati et al. [44] and Nouraei et al. [45] also mentioned that selection of inappropriate codes leads to a loss of income rather than making profits. Recoding or reassessing DRGs coding helps to generate potential income; however, it is time consuming and requires additional manpower to evaluate the coding. Our DL-based automated coding system can assist coders or physicians in selecting the best primary diagnosis for financial return. Moreover, physicians can select a secondary diagnosis from the top ten suggestions provided by our system.

Our study has several strengths. First, this is the first study to evaluate the DL performance for the prediction of DRGs. Second, the performance of our model was clinically satisfactory, which would help to reduce physicians’ workload, increase accurate coding for claiming reimbursement, increase hospital income, and improve hospital performance by using proper allocation of resources. There are several limitations that also need to be mentioned. First, this study only focuses on the urinary department. However, this is because the rate of coding error is high in the urinary department and the selection of appropriate DRGs coding is challenging. Second, while our model was internally validated, the performance of the model could vary if using other countries’ datasets. Third, our study did not evaluate how much money it would save or earn if the hospital chose to implement this model. However, the higher accuracy refers to the ability of our model to reduce the coding error of DRGs, which ultimately helps to save money, increase income, and improve the hospital performance for inpatient care.

## 6. Conclusions

This study revealed that a DL model, especially GRU, has the ability to predict DRGs’ primary diagnosis with high accuracy. Using this automated DRGs coding system would help to reduce incorrect coding, which can ultimately increase hospital income, ensure the fair allocation of medical resources, and improve hospital performance. Further studies are needed to evaluate the performance of the current model using data from other countries and to assess the financial benefits of this model.

## Figures and Tables

**Table 1:** Basic characteristics of patients included.

Variable	Number/Percentage
Total number of episodes	128,105
Total number of patients	81,486
Age range	
Age group	
0~20	4.51%
20~40	54.76%
40~60	42.79%
>60	0.02%
Gender	
Male	74.65%
Female	25.35%
Operation	
Yes	87.78%
No	12.22%
Additional diagnosis	
Yes	70.98%
No	29.02%
Procedure	
Yes	98.82%
No	1.18%
Drug	
Yes	99.58%
No	0.42%
Number of drugs input	461
Number of diseases input	200
Number of procedures input	1636
Number of operations input	927
Number of output	200

**Table 2:** Performance of deep learning models.

Model	Precision	Recall	F1-Score	Accuracy	AUROC	Ranking Loss
GRU	0.83	0.66	0.73	0.72	0.99	0.01
ANN	0.82	0.57	0.67	0.68	0.99	0.01

#Note: micro-AUROC.

**Table 3:** Sensitivity analysis.

Basic Info	Drug	Procedure	Operation	Additional ICD	Precision	Recall	F1-Score	Accuracy	Micro-AUC	Label Ranking Loss
V	V	V	V	V	0.83	0.65	0.73	0.726	0.99	0.01
V	V	V	V		0.76	0.60	0.67	0.671	0.99	0.01
V	V	V		V	0.70	0.31	0.43	0.481	0.97	0.03
V	V		V	V	0.55	0.42	0.47	0.465	0.92	0.06
V		V	V	V	0.81	0.56	0.66	0.632	0.98	0.03
V	V				0.08	0.02	0.04	0.059	0.79	0.16
V		V			0.26	0.04	0.07	0.211	0.92	0.08
V			V		0.52	0.33	0.41	0.373	0.88	0.09
V				V	0.01	0.005	0.006	0.026	0.75	0.19
V					0.001	0	0.001	0.006	0.73	0.21

**Table 4:** Evaluation of the performance of GRU for predicting primary diagnosis.

Example	Age	Sex	Original Primary Diagnosis	Predicted Primary Diagnosis	Top 5 Primary Diagnoses
Patient #1	20–40	Male	Calculus of ureter	Calculus of ureter	1. Calculus of ureter. 2. Calculus of kidney. 3. Urinary tract infection, site not specified. 4. Calculus in urethra. 5. Acute pyelonephritis without lesion of renal medullary necrosis.
Patient #2	20–40	Female	Calculus of kidney	Calculus of kidney	1. Calculus of kidney. 2. Acute pyelonephritis without lesion of renal medullary necrosis. 3. Urinary tract infection, site not specified. 4. Pyelonephritis, unspecified. 5. Renal colic.
Patient #3	20–40	Male	Malignant bladder neoplasm, part unspecified.	Malignant bladder neoplasm, part unspecified.	1. Malignant bladder neoplasm, part unspecified. 2. Malignant bladder neoplasm, lateral wall. 3. Malignant bladder neoplasm, other specified sites. 4. Neoplasms of unspecified nature, bladder. 5. Benign neoplasm of bladder.
Patient #4	20–40	Male	Malignant bladder neoplasm, other specified sites.	Malignant bladder neoplasm, part unspecified.	1. Malignant bladder neoplasm, part unspecified. 2. Neoplasms of unspecified nature, bladder. 3. Malignant bladder neoplasm, lateral wall. 4. Malignant bladder neoplasm, other specified sites. 5. Hematuria.
Patient #5	20–40	Male	Acute pyelonephritis without lesion of renal medullary necrosis.	Urinary tract infection, site not specified.	1. Urinary tract infection, site not specified. 2. Acute pyelonephritis without lesion of renal medullary necrosis. 3. Acute cystitis. 4. Hematuria. 5. Orchitis and epididymitis, other, without mention of abscess.
